# Epibionts dominate metabolic functional potential of *Trichodesmium* colonies from the oligotrophic ocean

**DOI:** 10.1038/ismej.2017.74

**Published:** 2017-05-23

**Authors:** Kyle R Frischkorn, Mónica Rouco, Benjamin A S Van Mooy, Sonya T Dyhrman

**Affiliations:** 1 Department of Earth and Environmental Sciences and the Lamont-Doherty Earth Observatory, Columbia University, Palisades, NY, USA; 2 Department of Marine Chemistry and Geochemistry, Woods Hole Oceanographic Institution, Woods Hole, MA, USA

## Abstract

*Trichodesmium* is a genus of marine diazotrophic colonial cyanobacteria that exerts a profound influence on global biogeochemistry, by injecting ‘new’ nitrogen into the low nutrient systems where it occurs. Colonies of *Trichodesmium* ubiquitously contain a diverse assemblage of epibiotic microorganisms, constituting a microbiome on the *Trichodesmium* host. Metagenome sequences from *Trichodesmium* colonies were analyzed along a resource gradient in the western North Atlantic to examine microbiome community structure, functional diversity and metabolic contributions to the holobiont. Here we demonstrate the presence of a core *Trichodesmium* microbiome that is modulated to suit different ocean regions, and contributes over 10 times the metabolic potential of *Trichodesmium* to the holobiont. Given the ubiquitous nature of epibionts on colonies, the substantial functional diversity within the microbiome is likely an integral facet of *Trichodesmium* physiological ecology across the oligotrophic oceans where this biogeochemically significant diazotroph thrives.

## Introduction

The colonial, diazotrophic cyanobacterium *Trichodesmium* has a cosmopolitan distribution throughout the tropical and subtropical oceans where it has a keystone role in oligotrophic ecosystems because of its ability to supply biologically available nitrogen through N_2_ fixation and fixed carbon through photosynthesis (Capone *et al.*, 1997). Models suggest that *Trichodesmium* N_2_ fixation accounts for roughly half of the total 100–200 Tg of biologically fixed N_2_ annually (Bergman *et al.*, 2013), a supply that fuels the uptake of carbon by the broader community of photoautotrophs and ultimately the export of carbon to the deep sea (Arrigo, 2005). In the oligotrophic oceans where *Trichodesmium* occurs, there is intense competition for resources such as phosphorus and iron, which can limit *Trichodesmium* N_2_ fixation (Sañudo-Wilhelmy *et al.*, 2001) and growth (Krishnamurthy *et al.*, 2007).

*Trichodesmium* cells grow as filaments, which aggregate to form colonies up to a millimeter in diameter, creating stable substrates that concentrate fixed carbon and nitrogen relative to surrounding seawater (Capone *et al.*, 1997). A hallmark of these colonies is their ubiquitous association with a diverse assemblage of microorganisms that are dominated by heterotrophic bacteria, as well as photosynthetic and even other N_2_ fixing bacteria (Sheridan *et al.*, 2002; Hmelo *et al.*, 2012; Momper *et al.*, 2014; Rouco *et al.*, 2016). Collectively, these tightly associated organisms are referred to as epibionts, and they form the *Trichodesmium* microbiome, a distinct community that is taxonomically different from planktonic microbes in surrounding seawater (Hmelo *et al.*, 2012).

Despite the global biogeochemical significance of *Trichodesmium* and the ubiquitous presence of a community of tightly associated microorganisms, eco-physiological studies of *Trichodesmium* have rarely considered this consortia of co-occurring organisms as a holobiont. The microbiome’s taxonomic diversity, functional diversity and the interplay between host and epibionts within the holobiont is only beginning to be explored (Hewson *et al.*, 2009; Van Mooy *et al.*, 2012; Rouco *et al.*, 2016), but could help explain the fundamental unknowns that persist regarding *Trichodesmium* distribution and activities across different environments. Exploring the metabolic functional potential contained within the *Trichodesmium* microbiome is a key step toward gaining a mechanistic understanding of how this relationship influences the fitness of the holobiont and subsequently the fate of fixed carbon and nitrogen in the oligotrophic ocean. Here we use metagenomic sequencing of *Trichodesmium* colonies collected from stations along a gradient of phosphorus in the western North Atlantic to examine microbiome composition, functional diversity and metabolic contributions to the holobiont.

## Materials and methods

### Field sampling

*Trichodesmium* colonies were collected with surface water net tows along a cruise transect in the western North Atlantic aboard the *R/V Atlantic Explorer* (AE1409) during May 2014. Sampling occurred at the same time each day (~0730–0830 hours) using nets with a mesh size of 130 μm. Nets were deployed and hauled through the surface water column six times before recovery, such that each sample represented thousands of liters of water. Individual *Trichodesmium* colonies were isolated and washed three times by successive transfer through fresh 0.2 μm sterile-filtered local surface seawater to remove all but tightly associated epibionts. A pooled sample of colonies was isolated and processed from each station. For each sample, an average of ~30 cleaned colonies were transferred onto 47 mm 5 μm pore size polycarbonate filters, gently vacuum filtered to remove excess liquid, flash frozen and stored in liquid nitrogen until extraction and sequencing. There were no discernable changes in average colony size from one station to another across the transect. In order to broadly assess the microbiome composition of the North Atlantic *Trichodesmium* populations, colony composition was sampled to reflect the distribution of *Trichodesmium* colony morphology found in net tows. At all stations, raft type colonies were much more abundant than puff or bowtie variants with approximately 30 rafts to 2 puff/bowtie colonies. As such, the data largely reflect the dominant raft morphology.

### Chemical analyses

Total dissolved phosphorus was determined on 0.2 μm filtrates of surface water (~5 m depth) samples collected by a Niskin rosette outfitted with conductivity, temperature, and depth sensors into acid-clean polycarbonate bottles. Samples were processed at the SOEST Laboratory for Analytical Biogeochemistry at the University of Hawaii, Honolulu, HI, USA, according to facility protocols. Alkaline phosphatase activity samples were obtained by placing 2–5 cleaned *Trichodesmium* colonies on 5 μm PC filters, gently vacuum filtering away excess liquid, then storing in 47 mm plastic Petri dishes at –20 °C until analysis. Samples were processed as previously described (Dyhrman and Ruttenberg, 2006) using 6,8-difluoro-4-methylumbeliferyl phosphate (DiMufP) on a Synergy H1 Hybrid plate reader using the Gen5 software package (BioTek, Winooski, VT, USA) (Dyhrman and Ruttenberg, 2006). N_2_ fixation was measured using the acetylene reduction technique as previously described (Capone, 1993; Paerl, 1994). Briefly, approximately 20 *Trichodesmium* colonies were placed in a 60 ml polycarbonate bottle containing 60 ml of filtered seawater. A 1 ml aliquot of acetylene was injected into the bottle through a septum cap, the bottle was gently inverted and allowed to incubate in an on-deck incubator at ambient temperature and light. The headspace of the bottle was analyzed for ethylene approximately every 30 min and the rate of ethylene production through acetylene reduction was determined by linear regression. All incubations were conducted in triplicate and harvested between approximately local noon and 1400 hours.

### DNA extraction and sequencing

Total genomic DNA was extracted from samples using the MoBio Power Plant Pro DNA Isolation Kit (MoBio Laboratories, Inc., Carlsbad, CA, USA) following the manufacturer instructions. An average concentration of 30 ng μl^–1^ of genomic DNA for each sample was sequenced at the Argonne National Lab (Lemont, IL, USA). Genomic DNA was quantified using the Invitrogen (Carlsbad, CA, USA) qubit and sheared using the Covaris Sonicator (Woburn, MA, USA) to the desired size range. Libraries were then generated using WaferGen’s Apollo324 automated library system and Illumina (San Diego, CA, USA) compatible PrepX ILMN DNA library kits following the manufacturer’s instructions. Resulting libraries were then size-selected using the Sage BluePippin (Beverly, MA, USA) and sequenced on one 2 × 100 bp lane of the Illumina HiSeq2000. During library preparation, an average insert size of approximately 750 base pairs (bp) was targeted. Metagenomic reads from the six samples are available on the NCBI Sequence Read Archive under BioProject number PRJNA330990.

### Sequence assembly and analysis

Metagenomic reads were first trimmed using Sickle with default settings (https://github.com/najoshi/sickle). Trimmed forward and reverse reads were then converted to fasta with the fq2fa command in IDBA-UD (Peng *et al.*, 2012). Reads from the six samples were assembled into scaffolds to create a merged assembly, using IDBA-UD under default parameters in order to yield robust assembly of the western North Atlantic *Trichodesmium* holobiont, modeling our approach after similar environmental metagenomic investigations (Handley *et al.*, 2012; Dombrowski *et al.*, 2016). This merged assembly of pooled colony metagenomic reads from across six stations was used for the bulk of the analyses presented herein.

Scaffolds produced by the merged assembly were clustered into genome bins by tetranucleotide frequency and read coverage of individual samples using MaxBin 2.0 set with default parameters (Wu *et al.*, 2015). Genome completeness was estimated at >65% using MaxBin, resulting in robust gene set comparisons for the majority of genome bins. Relative abundance estimates were calculated after Aylward *et al.* (2014) by multiplying the length of contigs in each bin by the number of reads recruited (coverage), then summing across genome bins. This method has shown good correlation with 16S-based results in other metagenomic data sets (Aylward *et al.*, 2014). Binned scaffolds were translated into predicted proteins using Prodigal on the metagenomic setting (Hyatt *et al.*, 2010). The resulting protein sequences were annotated using the blastp program of DIAMOND against the NCBI nr database (Suzek *et al.*, 2007; Buchfink *et al.*, 2015). The organismal identity of each bin was determined by assessing the nr database taxonomic affiliation of the best hits, with identity determined as the majority (>70%) taxonomic affiliation of predicted proteins in a bin. Proteins from each epibiont bin were also annotated against the SEED subsystems using the RAST online annotation program to assess differences in functional categories (Aziz *et al.*, 2008; Overbeek *et al.*, 2014). Although the majority of our analyses were based on the merged, six station metagenome assembly, we also examined regional difference in epibiont community structure by grouping northern (*n*=2) and southern (*n*=4) stations as replicates. Variance in this north–south epibiont community structure (species relative abundance) was visualized using principal component analysis in R (www.r-project.org), using the rda function in the vegan package (https://github.com/vegandevs/vegan) (Oksanen *et al.*, 2015). A permutational multivariate analyses of variance test (*P*<0.1) was performed to examine differences in community structure between the two northern stations versus the four southern stations, using a Bray–Curtis dissimilarity distance matrix (Anderson, 2001). Unpaired *t*-tests were calculated using GraphPad (La Jolla, CA, USA).

To prepare for orthologous group (OG) clustering, the six individual station assemblies were translated into predicted proteins as described above and merged together. These proteins were then filtered to remove sequences <70 amino acids and clustered into OGs by performing a reciprocal blastp with DIAMOND and then using MCL (Markov cluster algorithm), set to an inflation parameter of 1.4 as previously described (Bertrand *et al.*, 2015). Taxonomic composition of the individual station assembly OG clustered proteins was determined by DIAMOND blastp using the previously assembled genome bins as a reference. OGs were classified as ‘epibiont only’ if no genes making up the group had best blast hits to the four *Trichodesmium* genome bins or to proteins that are taxonomically affiliated with *Trichodesmium*. OGs defined as ‘both’ were composed of epibiont and *Trichodesmium* identified proteins.

Functional annotation of the predicted proteins clustered into OGs followed a tiered protocol. First, the size filtered and clustered proteins were annotated using blastp search with DIAMOND against the UniRef90 database (Buchfink *et al.*, 2015) and the Kyoto Encyclopedia of Genes and Genomes (KEGG) with the online Automatic Annotation Server using the single-directional best-hit method targeted to prokaryotes and with the metagenomic option selected. Single functional annotations for entire OGs were determined by taking the majority annotation for all proteins clustered into that group. To refine the annotations of select proteins, curated databases and protein models were used. The alkaline phosphatase-identified OG annotations were refined by performing DIAMOND searches against representative proteins from COGs 3211 (PhoX), 1785 (PhoA) and 3540 (PhoD) (Luo *et al.*, 2009), as well as the protein sequences of three previously identified putative alkaline phosphatases in the IMS101 genome (PhoA: YP723031, PhoX: YP723360, and PhoX2: YP723924) (Orchard *et al.*, 2009). Putative alkaline phosphatase metal cofactors were determined based on previous investigations (Luo *et al.*, 2009; Rodriguez *et al.*, 2014; Yong *et al.*, 2014). PepM OGs were obtained by DIAMOND blast against representative proteins from PFAM 13714 (PEP_mutase). Blast results were accepted if the e-value was <1 × 10^−5^ with a bit score >50. For comparisons against the putative PhoA gene from IMS101, OGs within the metagenomes were considered homologous to this protein if they passed the blast requirements above, and contained UniRef blast homologs to the YP723031 gene from *T. erythraeum* IMS101 and other putative alkaline phosphatase genes identified through KEGG or UniRef annotation.

## Results and discussion

### *Composition of the* Trichodesmium *holobiont*

**Figure 1:**
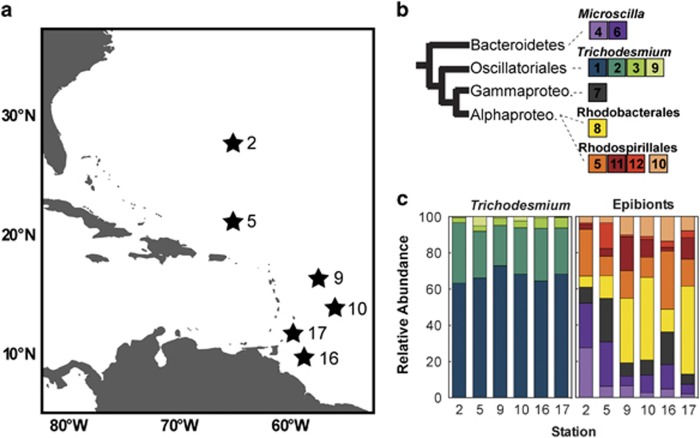
Sampling locations, genome bin identity and relative community composition of *Trichodesmium* holobiont members in the western North Atlantic. (**a**) *Trichodesmium* colonies were collected from near surface water using hand held nets at six stations during spring 2014 in the western North Atlantic. (**b**) Taxonomic affiliations of the 12 genome bins generated from a merged metagenome assembly and represented on a simplified phylogenetic tree to the class level. Gammaproteo., Gammaproteobacteria. Alphaproteo., Alphaproteobacteria. (**c**) The relative community composition of the holobiont members along the transect, noting that values were determined using reads from single samples of pooled colony metagenomic libraries. Values were calculated by multiplying contig lengths in each bin by read mapping coverage, following Aylward *et al.* (2014).

High-throughput paired-end sequencing (Supplementary Table 1) was performed on total genomic DNA extracted from *Trichodesmium* colonies collected at six stations in the western North Atlantic (Figure 1a). A merged genomic DNA assembly of sequences from all stations was used to identify 12 unique taxonomic genome bins conserved across all 6 North Atlantic stations, 9 of which were estimated to be over ~65% complete based on single copy marker gene presence (Supplementary Figure 1), results that are similar to recoveries from other environmental metagenomic data sets (Handley *et al.*, 2012; Dombrowski *et al.*, 2016). All bins were identified down to the lowest definitive taxonomic level possible. Of the 12 taxonomic bins, there were 4 identified as *Trichodesmium*, which suggests that multiple *Trichodesmium* species could be present in these samples. Consistent with this observation, analyses of surface water and colonies along a similar transect in the western North Atlantic detected species from at least three co-occurring clades of *Trichodesmium* in this region (Rouco *et al.*, 2014, 2016). The remaining eight genomic bins were identified as heterotrophic epibionts including two in the Bacteroidetes genus *Microscilla,* one Gammaproteobacterium, one Alphaproteobacterium in the order Rhodobacterales and four Alphaproteobacteria in the order Rhodospirillales (Figure 1b). These results contribute new information about the *Trichodesmium* microbiome, building upon a previous 16S clone library survey of epibiont diversity from the North Atlantic (Hmelo *et al.*, 2012), recent high-throughput assessment of epibiont taxonomic diversity across three ocean basins (Rouco *et al.*, 2016), and metatranscriptomic profiles of *Trichodesmium* communities from the South Pacific (Hewson *et al.*, 2009). Similarities in the taxonomic groups dominating samples in all of these aforementioned studies indicate that the epibiont metagenomes detected here are likely from core members of the *Trichodesmium* holobiont. In addition, this microbiome community does not merely represent general particle-associated microbes, as the community recovered here was distinct from those previously found on sinking particles, which have been shown to be enriched with Deltaproteobacteria, Planctomyces and Bacteroidetes in genera other than *Microscilla* (Fontanez *et al.*, 2015).

**Figure 2:**
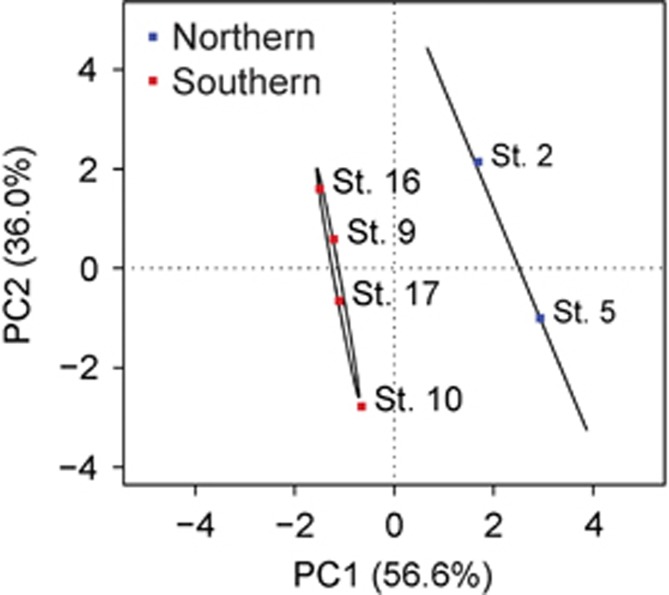
Principal component analysis of the relative abundance of *Trichodesmium* microbiome members. The 95% confidence intervals between northern and southern stations are indicated by black ellipses. The relative abundance of epibionts in the microbiome were significantly (*P*<0.1) different between the two northern and four southern stations (permutational multivariate analyses of variance *P=*0.067).

To estimate changes in relative abundance of holobiont members across the cruise transect, reads from each station were mapped to the taxonomic bins after Aylward *et al.* (2014). All microbiome members were detected at each station, confirming that these epibionts represent core components of the microbiome, although the relative abundance was variable from station to station (Figure 1c). Using the relative abundance of different epibiont members, microbiome communities clustered according to their sampling site, with the two northernmost stations separated from the southernmost stations along the PC1 axis of a principal component analysis (Figure 2). A permutational multivariate analyses of variance analysis (*P*=0.067), confirmed a differential community structure between the two northern and four southern stations. This may be driven in part by the abundance of *Microscilla* (bins 4 and 6), which was significantly different (*t*-test, *P*=0.010) between the two northern stations and the four southern stations. There were no significant differences between the northern and southern stations in N_2_ fixation rate, colony PO_4_ turnover or alkaline phosphatase activity, however, the two northernmost stations had significantly lower total dissolved phosphorus compared with the four southern stations (0.08 and 0.2 μM total dissolved phosphorus, respectively, *t-*test, *P*=0.0157; Supplementary Table 1). As such, *Microscilla* relative abundance was higher at the stations with increased oligotrophy. Although further work measuring more parameters over a greater range of conditions is warranted to address the consistency of these relationships, the differential distribution of members of the core microbiome between regions suggests that variations in epibiont community structure could be modulated by the geochemical environment.

**Figure 3:**
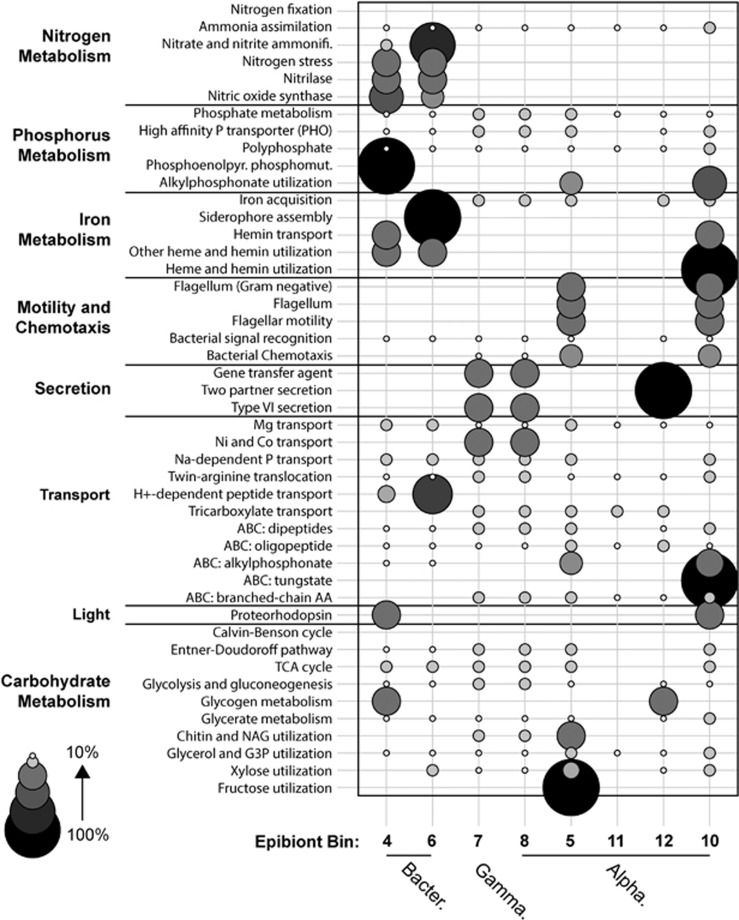
Enrichment of functional pathways recovered from epibiont genome bins. The distribution is based on RAST annotation against the SEED subsystems (Aziz *et al.*, 2008; Overbeek *et al.*, 2014). The contribution of each epibiont to a given SEED subsystem is scaled relative to the percentage of genes within each subcategory found in each genome bin. Ammonifi., Ammonification; ABC, ABC transporter; AA, amino acid. Alpha., Alphaproteobacteria; Bacter., Bacteroidetes; Gamma., Gammaproteobacterium.

In order to examine the diversity of metabolic pathways in the *Trichodesmium* microbiome, the genome bins were functionally annotated against the SEED subsystems to assess differences in broad functional categories between epibionts (Overbeek *et al.*, 2014). This functional analysis showed that while key capacities like ammonia assimilation, phosphate metabolism, transport of organic compounds and aerobic metabolic pathways like the tricarboxylic acid cycle were largely uniformly present in all epibionts, there were certain SEED subsystems that were enriched or uniquely present in discrete groups (Figure 3). The *Microscilla* genome bins were enriched in functional categories related to nitrogen metabolism, such as nitrate, and nitrogen stress functions, as well as the synthesis and utilization of reduced phosphorus compounds (Figure 3), which could help explain their relative abundance at the most oligotrophic stations (2 and 5) (Supplementary Table 1). The Gammaproteobacterium and Rhodobacterales (bins 7 and 8) were enriched with functions related to the uptake and exchange of genetic information (gene transfer agents and bacterial secretion systems), whereas the Rhodospirillales bins were enriched in motility-related functions, the uptake of tungstate and the utilization of plant-derived sugars like fructose (Figure 3). In marine bacteria, secretion systems and motility functions have been implicated in the transfer of toxins between adjacent cells and pathogenicity (Salomon *et al.*, 2015) and the modulation of these activities could influence relationships between epibionts or the nature of the host–microbiome relationship. Finally, homologs to genes encoding the light-mediated proton pump proteorhodopsin were found in *Microscilla*, as well as the Rhodospirillales (Figure 3). In sum, these data show that there are differences in functional metabolic capacity between epibiont groups present in the core microbiome.

Iron and phosphorus-related SEED subsystems within the microbiome (Figure 3), like siderophore and heme-related functions, or reduced phosphorus utilization pathways, may be particularly critical to *Trichodesmium* physiological ecology given that iron and phosphorus are known drivers of *Trichodesmium* activities in the study region (Sañudo-Wilhelmy *et al.*, 2001; Chappell *et al.*, 2012). The relative proportion of microbiome genome bins in which key marker genes were found for phosphonate metabolism, heme and siderophore utilization, were compared with free-living microbial communities in the Sargasso Sea region of the western North Atlantic. The *Trichodesmium* microbiome was enriched nearly twofold in the phosphonate utilization marker *phnJ* relative to genome equivalents in the Global Ocean Survey data set from the Sargasso Sea (Karl *et al.*, 2008) (Supplementary Table 2). Phosphonates are known to be an important source of bioavailable phosphorus for *Trichodesmium* in the North Atlantic (Dyhrman *et al.*, 2006) and the enrichment of *phnJ* in the microbiome indicates the importance of this bond class of phosphorus to the holobiont relative to free-living microbes in the upper water column. Heme transporters and siderophore/vitamins transporters were present in all core microbiome genome bins, relative to communities of free-living Sargasso Sea microbes in which these functions were present in roughly 2% and 18% of genome equivalents, respectively (Tang *et al.*, 2012; Supplementary Table 2). This enrichment is again suggestive of the importance of iron to the holobiont relative to free-living microbes in the upper water column. The apparent enrichment of phosphorus and iron functions in the microbiome may provide a competitive advantage to the holobiont relative to co-occurring free-living microbes and underlie possible syntrophic interactions between *Trichodesmium* and the microbiome, but this enrichment could also enhance internal competition within the holobiont. Taken together, the taxonomic and functional diversity within the *Trichodesmium* microbiome, and its variation along the transect, raises the possibility that the fitness of holobiont members or regional biogeochemistry could influence the distribution and activities of the holobiont.

### The microbiome dominates holobiont functional diversity

**Figure 4:**
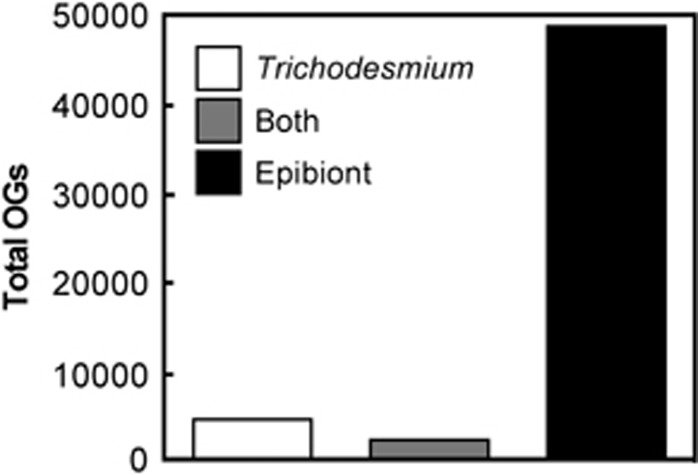
Distribution of OGs in the *Trichodesmium* holobiont. A total of 264 073 predicted proteins (>70 amino acids) were clustered into 55 738 OGs. OGs were considered ‘*Trichodesmium* only’ or ‘epibiont only’ if they were composed of predicted proteins solely from those organisms. The ‘both’ category refers to OGs composed of predicted proteins from *Trichodesmium* and epibiont genome bins.

We evaluated the distribution of and diversity of physiological capabilities in the *Trichodesmium* host (genome bins 1–3 and 9) compared with members of their microbiome by functionally analyzing OGs of proteins. The taxonomic composition of predicted proteins in OGs was assessed to investigate the extent to which the *Trichodesmium* microbiome augments the global metabolic potential of the holobiont. Of the total 55 738 unique OGs, 4546 OGs were composed solely of predicted proteins identified as *Trichodesmium* and 2252 OGs were composed of predicted proteins from *Trichodesmium* and epibiont genome bins (Figure 4). The *Trichodesmium* spp. present in the field samples contained over two times the number of gene families (6798) present in *T. erythraeum* IMS101 genome, which is predicted to encode 5076 proteins (Walworth *et al.*, 2015) and yielded 2982 OGs following the clustering protocol described herein. *Trichodesmium* does not exhibit genome streamlining like other oligotrophic cyanobacteria (Walworth *et al.*, 2015), and the disparity in the number of OGs between type strain and field samples could be driven by the presence of multiple species with varying gene contents, or the fact that *T. erythraeum* is not common in *Trichodesmium* populations in the North Atlantic (Rouco *et al.*, 2014).

Given the taxonomic diversity of the microbiome, we would expect the number of epibiont OGs to exceed that of *Trichodesmium.* Nearly 90% of the total 55 738 OGs were composed only of epibiont-identified proteins (Figure 4). This number exceeds what would be expected from the eight core microbiome members together and likely comes from less abundant epibionts that were not sequenced deeply enough to yield genome bins. These epibiont-only OGs represent functions without homologs in *Trichodesmium,* suggesting recruitment and selection in the microbiome could expand and alter the metabolic repertoire of the holobiont. To more conservatively examine functional content, OGs were assigned functional annotations using KEGG, and the epibiont-only OGs were found to have twice as many unique functions relative to those shared between epibionts and *Trichodesmium,* and nearly 10 times as many unique functions as those found in *Trichodesmium* alone (Supplementary Figure 2). These differences are most notable within the genetic information processing module, consistent with the broad taxonomic diversity within the microbiome and to some extent in the carbohydrate and lipid metabolism module (Supplementary Figure 2), which is represented by an abundance of carbohydrate active enzymes not present in *Trichodesmium* (see below).

**Figure 5:**
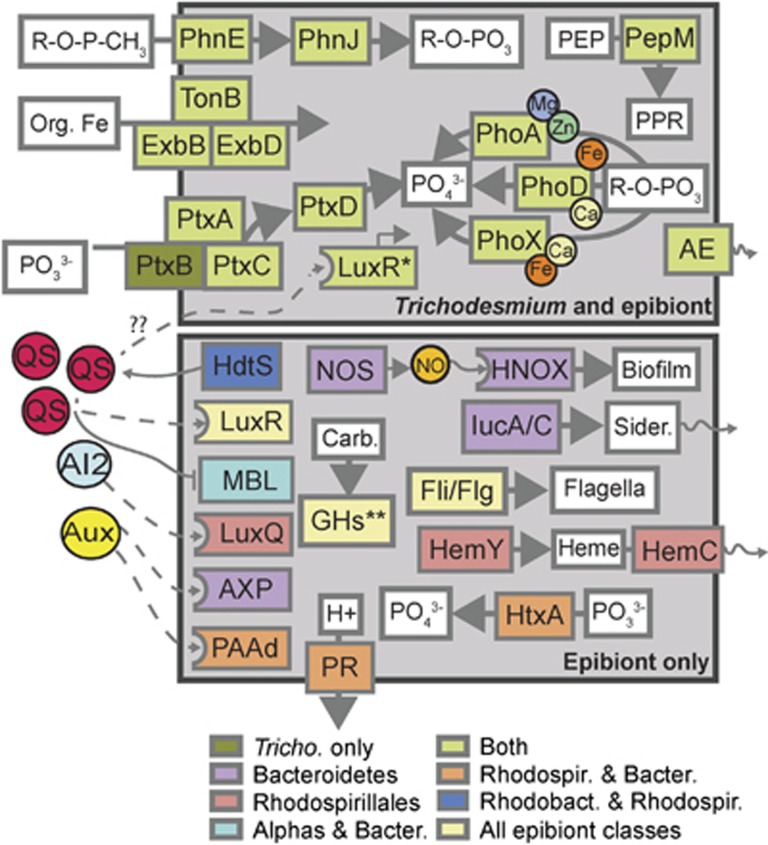
Cell diagram depicting OGs with key functions shared across the *Trichodesmium* holobiont, or unique to the microbiome. *Trichodesmium* LuxR is homologous only in DNA-binding region (*) and the GH protein represents many enzymes with specific targets unique to epibionts (**) (Supplementary Table 3). The ‘all epibiont’ category refers to homologs that were found in all epibiont genome bins. AE, auxin efflux protein; AI2, autoinducer 2; Aux, auxin; AXP, auxin-regulated protein; Fli/Flg, flagella biosynthesis and motility; GH, glycoside hydrolase; Carb., Carbohydrate; HemY, HemC, heme group synthesis and export; HNOX, heme-nitric oxide/oxygen binding protein; HtxA, alternative phosphite dehydrogenase; HdtS, AHL synthase; IucA/C, siderophore biosynthesis; Sider., Siderophore; LuxQ, AI2 transcriptional activator; LuxR, QS transcriptional activator; MBL, Metallo-beta lactamase; NO, nitric oxide; NOS, nitric oxide synthase; PhnE, phosphonate transporter inner membrane subunit; PhnJ, C-P lyase; PepM, phosphoenolpyruvate mutase; PEP, phosphoenolpyruvate; PPR, 3-phosphonopyruvate; PR, proteorhodopsin; PtxABC, phosphite transport system; PtxD, phosphite dehydrogenase; PhoA, PhoD, PhoX, alkaline phosphatases with putative metal cofactors (Mg, magnesium, Zn, zinc, Ca, calcium, Fe, iron); PAAd, phenylacetic acid degrading protein; QS, quorum sensing molecule; TonB-ExbB-ExbD, putative organic iron uptake system. *Tricho*., *Trichodesmium*; Alphas, Alphaproteobacteria; *Micros*., *Microscilla*; Rhodospir., Rhodospirillales; Rhodobact., Rhodobacterales.

Specific OGs enriched or unique to the microbiome included proteins with functions related to cell–cell signaling and the processing of organic matter (Figure 5). Homologs to the proteins responsible for sensing and responding to quorum-sensing molecules like acyl homoserine lactone (AHL), the LuxR family, were detected in all epibionts (Figure 5). Although putative LuxR homologs identified as belonging to *Trichodesmium* genome bins shared sequence identity along the DNA-binding domain, it has been previously determined that these *Trichodesmium* genes do not share the characterized AHL-binding residues of these proteins (Vannini *et al.*, 2002; Patankar and Gonzalez, 2009; Van Mooy *et al.*, 2012). This indicates that *Trichodesmium* is either not involved in quorum sensing, or that the LuxR variant present in *Trichodesmium* is responding to a different quorum-sensing molecule. Homologs to proteins in the LuxQ family, which is part of a sensor kinase complex that detects the autoinducer-2 (AI-2) signaling molecule (Miller and Bassler, 2001) were detected in the Rhodospirillales (bins 5 and 11) (Figure 5). Homologs of LuxI, a gene responsible for synthesis of AHL quorum-sensing molecules, and a homolog to acyl-sn-glycerol-3-phosphate acyltransferase, a putative AHL synthase designated HdtS and characterized in *Pseudomonas fluorescens* (Case *et al.*, 2008), were found in the Rhodobacterales and Rhodospirillales epibionts (Figure 5). Quorum-sensing circuits, and subsequently the activities they modulate, can be broken through the secretion of molecules that break down AHLs. This process, termed quorum quenching, can be driven by quorum quenching enzymes like metallo-beta-lactamases, which degrade antibiotics and AHLs (Hong *et al.*, 2012; Fetzner, 2014). Homologs of this enzyme were detected in *Microscilla,* Rhodospirillales and Rhodobacterales (Figure 5). Taken together, these data indicate that a suite of different quorum-sensing pathways are present within members of the microbiome. In fact, quorum-sensing molecules and quorum-quenching activity have been detected in *Trichodesmium* colonies from environmental samples, and quorum-sensing molecules added to sinking particles from *Trichodesmium* rich environments stimulate the hydrolysis of organic matter (Hmelo *et al.*, 2011; Van Mooy *et al.*, 2012; Krupke *et al.*, 2016). The concordance between metagenomic and *in situ* chemical evidence of these pathways constitutes strong evidence that quorum sensing is active in *Trichodesmium* colonies, likely regulates a range of microbiome functions, and mediates interactions between host and microbiome.

OGs with homologs of nitric oxide synthase (NOS) and the sensing protein (H-NOX) were found in both *Microscilla*-identified genome bins (Figure 5). The presence of both pathways have only previously been detected in an Alphaproteobacterial lineage (Rao *et al.*, 2015), and their presence here in *Microscilla,* a genus in the Bacteroidetes class, suggests that the ability to both produce and sense NO is more widespread in marine bacteria than previously thought. The NO signaling pathway can be triggered by *Trichodesmium* to induce biofilm formation (Rao *et al.*, 2015), and concomitant quorum sensing is used to modulate a range of responses including N_2_ fixation, siderophore and enzyme biosynthesis, as well as motility, aggregation and biofilm formation in other systems (Bassler, 2012).

Finally, homologs were detected for the efflux and sensing of auxin, a family of plant hormones implicated in stimulating a suite of plant processes, including growth and division, which can be synthesized by both plants and bacteria (Spaepen and Vanderleyden, 2011) (Figure 5). In the microbiome, the *Microscilla* epibionts contained an auxin responsive gene, and both *Microscilla* and the Rhodospirillales contained genes for the degradation of phenylacetic acid (Figure 5), an auxin-like compound produced by certain plant-associated bacteria with known antimicrobial activity (Somers *et al.*, 2005). For heterotrophic bacteria, auxin compounds have been shown to induce resistance to stress agents and biofilm formation, upregulation of the tricarboxylic acid cycle and amino-acid biosynthesis, and increased enzyme activity (Spaepen and Vanderleyden, 2011 and references therein). The known roles of auxin, NO and AHLs in cell–cell signaling suggests that these gene targets could be used to query potential interactions, such as mutualism or parasitism within the holobiont. Overall, the presence and known activation of a number of different signaling pathways in the *Trichodesmium* microbiome suggests that physiological activities may be modulated and coordinated within the holobiont, and could vary depending on the relative abundance of epibionts and with fluctuations in taxonomic composition of the microbiome or the fitness of the *Trichodesmium* host.

Another set of OGs enriched in the microbiome were identified as members of the glycoside hydrolase (GHs) family of carbohydrate active enzymes (CAZymes). The majority of GH OGs were unique to epibionts with over 80% (21 out of 26) of the unique GH-identified OGs composed solely of epibiont proteins (Supplementary Table 3). These GHs are predicted to be active against a diverse suite of compounds including xylans, lichenin, chitin, xylose and arabinose (Supplementary Table 3). GH enzymes have been implicated in the bacterial processing of algal-derived polysaccharides (Teeling *et al.*, 2012) and the processing of *Trichodesmium* exudates by epibionts has been suggested to affect the rate of carbon and nitrogen transfer to the deep ocean (Herbst and Overbeck, 1978; Nausch, 1996; Hmelo *et al.*, 2012). As such, microbiome metabolism and its potential modulation through cell–cell signaling, may influence organic matter processing within the holobiont, competition for resources, and the concomitant impact on the fate of carbon and nitrogen. The abundance of these GHs and other epibiont-only OGs representing key functions highlights how the microbiome of *Trichodesmium* expands holobiont functional diversity. The extent to which this metabolic potential increases or decreases host fitness may influence the cycling of both nitrogen and carbon.

### Resource niche partitioning in the *Trichodesmium* holobiont

Iron and phosphorus bioavailability have been shown to influence the distribution and activities of diazotrophic communities (Deutsch *et al.*, 2007; Krishnamurthy *et al.*, 2007), and both are major drivers of *Trichodesmium* abundance and N_2_ fixation rate in the Atlantic (Wu *et al.*, 2000; Sañudo-Wilhelmy *et al.*, 2001; Sohm and Capone, 2006; Webb *et al.*, 2007; Moore *et al.*, 2008, 2009). To evaluate the contribution of the microbiome to potential iron and phosphorus cycling in the holobiont, OGs were additionally screened for the presence of key processes including the metabolism of organic phosphorus, processing of reduced phosphorus, and metabolism and transport of organic iron. The alkaline phosphatase enzyme hydrolyzes phosphate from phosphoester bond dissolved organic phosphorus, and is central to microbial phosphorus bioavailability in the western North Atlantic (Mahaffey *et al.*, 2014), where dissolved organic phosphorus concentration is higher than the inorganic phosphate concentration (Lomas *et al.*, 2010). Homologs for the alkaline phosphatases PhoA, PhoD and PhoX were found in both *Trichodesmium* and the microbiome (Figure 5). PhoX was found in *Trichodesmium*, as well as all epibionts with the exception of one Rhodospirillales (bin 12). Within the core microbiome, PhoX was more common than PhoA, which was only found in bins 5, 6, 7 and 10, and PhoD, which was only detected in the *Microscilla* genome bins (bins 4 and 6). This observation agrees with previous molecular surveys that found PhoX to be more prevalent than PhoA among oligotrophic planktonic marine bacteria (Sebastian and Ammerman, 2009).

OGs with proteins for the uptake and metabolism of reduced phosphorus were also present in both *Trichodesmium* and the microbiome (Figure 5). The phosphite dehydrogenase PtxD was detected in *Trichodesmium* and the microbiome, and a homolog of an alternate phosphite dehydrogenase enzyme was detected in the Rhodospirillales and in *Microscilla* in the form of HtxA, a gene characterized in *Pseudomonas stutzeri* that has a similar function to PtxD (Metcalf and Wolfe, 1998) (Figure 5). When paired to the ABC transporter PtxABC, PtxD allows *Trichodesmium* to use phosphite as a sole phosphorus source (Polyviou *et al.*, 2015). In addition, *Trichodesmium* consortia at station 9 took up radiolabeled phosphite (Van Mooy *et al.*, 2015) and the data herein suggest that phosphite is a substrate for both the *Trichodesmium* and multiple members of the microbiome, which could contribute to resource competition between holobiont members.

Other pathways to metabolize reduced phosphorus, like those involved in phosphonate transport (Phn E) and hydrolysis were detected in both *Trichodesmium* and the microbiome. PhnJ, a component of the broad specificity C-P lyase, was found in *Trichodesmium*, consistent with its presence in the *T. erythraeum* IMS101 genome (Dyhrman *et al.*, 2006), two Rhodospirillales epibionts and the Gammaproteobacterium (Figure 5). Finally, phosphoenolpyruvate mutase (PepM), the enzyme that catalyzes formation of a carbon–phosphorus bond through the conversion of phosphoenolpyruvate to phosphonopyruvate and thought to be a major source of biological phosphonate production (Metcalf and van der Donk, 2009), was detected in *Microscilla* bin 4, an unbinned epibiont species, as well as one of the *Trichodesmium* genome bins (Figure 5). Van Mooy *et al.* (2015) showed elevated rates of phosphonate compound biosynthesis at station 5, and the metagenome findings suggest that phosphonate biosynthesis may be driven by both *Trichodesmium* and members of the microbiome, in particular *Miroscilla* in this system. Overall, the abundance and diversity of phosphorus-related functions shared between *Trichodesmium* and the microbiome is consistent with the importance of active phosphorus cycling within the holobiont, as may be expected given the known role of phosphorus in limiting *Trichodesmium* populations in this region (Sañudo-Wilhelmy *et al.*, 2001; Dyhrman *et al.*, 2002).

Similar to phosphorus-related OGs, iron-related OGs were also enriched in the holobiont (Supplementary Table 2), however, the majority of these were present only in epibionts, and with subtle differences in specific genes contributing to each OG between different members of the microbiome (Figure 5). Homologs to a siderophore biosynthesis protein were found exclusively in one *Microscilla* (bin 6) (Figure 5). In addition, homologs to heme group biosynthesis and exporter proteins were made up exclusively from those found within the *Microscilla* and Rhodospirillales (Figure 5). Homologs of the TonB-dependent transporter system predicted to transport heme- and siderophore-bound iron, were detected in *Trichodesmium* and all epibiont genome bins (Figure 5; Supplementary Table 2), suggesting that while not all holobiont members produce these molecules, they could be taken up and potentially utilized by the core microbiome and *Trichodesmium*. *Trichodesmium* is rarely maintained in axenic culture, so direct comparisons of iron uptake between epibionts and the host are challenging. Roe *et al.* (2012) found that siderophore-bound iron was not as accessible to the *Trichodesmium* holobiont as inorganic forms, at least relative to select epibiont isolates, which could readily utilize most iron sources. However, uptake of different iron forms has been shown to vary between colonies (Achilles *et al.*, 2003), suggestive of the fact that alterations in epibiont composition could influence the accessibility of different iron forms to the holobiont, and could increase competition for this nutrient in low iron, oligotrophic conditions. Overall, the production and uptake of different organic iron complexes by epibionts could shape the dynamics of iron-dependent processes like N_2_ fixation, particularly in the many low iron regions of the ocean. In contrast to the redundant phosphorus-related functions, the epibiont-only iron functions highlight how the microbiome contributes diverse and unique functions that have the potential to uniquely modulate the geochemical microenvironment within colonies. Iron is relatively more abundant in the study region than in other areas like the North Pacific (Sohm *et al.*, 2011 and references therein), and additional surveys of the functional composition of holobiont metagenomes from diverse ocean regimes would identify the consistency of the iron and phosphorus-related OG distributions within the *Trichodesmium* holobiont.

## Conclusions

The importance of microbiomes to marine metazoans is well established (for example, Hentschel *et al.*, 2012), but the role of the *Trichodesmium* microbiome in shaping the distribution, activities and concomitant biogeochemical impact of these consortia is still in its infancy. Here we show that the substantial majority of the metabolic potential in the *Trichodesmium* holobiont is contained within the microbiome. This finding suggests that within the microbiome, there is a palette of functional diversity that could modulate host fitness and subsequent biogeochemical impact across different environments. This study also provides an annotated holobiont metagenome that could serve as a template for future metatranscriptomic and metaproteomic investigations focused on tracking physiological activities or interactions within the holobiont. The microbiome may underpin *Trichodesmium*’s success in oligotrophic systems and could be an important facet determining its resilience in a future ocean that is likely to bring increased oligotrophic conditions (Riebesell *et al.*, 2009). Overall, these results suggest that *Trichodesmium* should not be considered in isolation, but rather studied as a dynamic microbial holobiont.

## References

[bib1] Achilles KM, Church TM, Wilhelm SW, Luther GWI, Hutchins DA. (2003). Bioavailability of iron to *Trichodesmium* colonies in the western subtropical Atlantic Ocean. Limnol Oceanogr 48: 2250–2255.

[bib2] Arrigo KR. (2005). Marine microorganisms and global nutrient cycles. Nature 437: 343–348. 16163345 10.1038/nature04159

[bib3] Anderson MJ. (2001). A new method for non-parametric multivariate analysis of variance. Austral Ecology26: 32–46..

[bib4] Aylward FO, Suen G, Biedermann PHW, Adams AS, Scott JJ, Malfatti SA et al. (2014). Convergent bacterial microbiotas in the fungal agricultural systems of insects. MBio 5: e02077. 25406380 10.1128/mBio.02077-14 PMC4251994

[bib5] Aziz RK, Bartels D, Best AA, DeJongh M, Disz T, Edwards RA et al. (2008). The RAST Server: rapid annotations using subsystems technology. BMC Genomics 9: 75. 18261238 10.1186/1471-2164-9-75 PMC2265698

[bib6] Bassler BL. (2012). Microbes as menaces, mates & marvels. Daedalus 141: 67–76.

[bib7] Bergman B, Sandh G, Lin S, Larsson J, Carpenter EJ. (2013). *Trichodesmium*—a widespread marine cyanobacterium with unusual nitrogen fixation properties. FEMS Microbiol Rev 37: 286–302. 22928644 10.1111/j.1574-6976.2012.00352.x PMC3655545

[bib8] Bertrand EM, Mccrow JP, Moustafa A, Zheng H, Mcquaid JB, Delmont TO et al. (2015). Phytoplankton – bacterial interactions mediate micronutrient colimitation at the coastal Antarctic sea ice edge. Proc Natl Acad Sci USA 112: 9938–9943. 26221022 10.1073/pnas.1501615112 PMC4538660

[bib9] Buchfink B, Xie C, Huson DH. (2015). Fast and sensitive protein alignment using DIAMOND. Nat Methods 12: 59–60. 25402007 10.1038/nmeth.3176

[bib10] Capone DG. (1993) Determination of nitrogenase activity in aquatic samples using the acetylene reduction method. In: Kemp PF, Sherr BF, Sherr EB, Cole JJ (eds). Handbook of Methods in Aquatic Microbial Ecology. CRC Press: Boca Raton, FL, USA.

[bib11] Capone DG, Zehr JP, Paerl HW, Bergman B, Carpenter EJ. (1997). *Trichodesmium*, a globally significant marine cyanobacterium. Science 276: 1221–1229.

[bib12] Case RJ, Labbate M, Kjelleberg S. (2008). AHL-driven quorum-sensing circuits: their frequency and function among the Proteobacteria. ISME J 2: 345–349. 18273067 10.1038/ismej.2008.13

[bib13] Chappell PD, Moffett JW, Hynes AM, Webb EA. (2012). Molecular evidence of iron limitation and availability in the global diazotroph *Trichodesmium*. ISME J 6: 1728–1739. 22402399 10.1038/ismej.2012.13 PMC3498915

[bib14] Deutsch C, Sarmiento JL, Sigman DM, Gruber N, Dunne JP. (2007). Spatial coupling of nitrogen inputs and losses in the ocean. Nature 445: 163–167. 17215838 10.1038/nature05392

[bib15] Dombrowski N, Donaho JA, Gutierrez T, Seitz KW, Teske AP, Baker BJ. (2016). Reconstructing metabolic pathways of hydrocarbon-degrading bacteria from the Deepwater Horizon oil spill. Nat Microbiol 1: 1–8. 10.1038/nmicrobiol.2016.57 27572965

[bib16] Dyhrman ST, Webb EA, Anderson DM, Moffett JW, Waterbury JB. (2002). Cell-specific detection of phosphorus stress in *Trichodesmium* from the western North Atlantic. Limnol Oceanogr 47: 1832–1836.

[bib17] Dyhrman ST, Chappell PD, Haley ST, Moffett JW, Orchard ED, Waterbury JB et al. (2006). Phosphonate utilization by the globally important marine diazotroph *Trichodesmium*. Nature 439: 68–71. 16397497 10.1038/nature04203

[bib18] Dyhrman ST, Ruttenberg KC. (2006). Presence and regulation of alkaline phosphatase activity in eukaryotic phytoplankton from the coastal ocean: implications for dissolved organic phosphorus remineralization. Limnol Oceanogr 51: 1381–1390.

[bib19] Fetzner S. (2014). Quorum quenching enzymes. J Biotechnol 201: 2–14. 25220028 10.1016/j.jbiotec.2014.09.001

[bib20] Fontanez KM, Eppley JM, Samo TJ, Karl DM, DeLong EF. (2015). Microbial community structure and function on sinking particles in the North Pacific Subtropical Gyre. Front. Microbiol 6: 1–14. 26042105 10.3389/fmicb.2015.00469 PMC4436931

[bib21] Handley KM, VerBerkmoes NC, Steefel CI, Williams KH, Sharon I, Miller CS et al. (2012). Biostimulation induces syntrophic interactions that impact C, S and N cycling in a sediment microbial community. ISME J 7: 800–816. 23190730 10.1038/ismej.2012.148 PMC3603403

[bib22] Hentschel U, Piel J, Degnan SM, Taylor MW. (2012). Genomic insights into the marine sponge microbiome. Nat Rev Microbiol 10: 641–654. 22842661 10.1038/nrmicro2839

[bib23] Herbst V, Overbeck J. (1978). Metabolic coupling between the alga *Oscillatovia redekei* and accompanying bacteria. Naturwissenschaften 65: 64–65.

[bib24] Hewson I, Poretsky RS, Dyhrman ST, Zielinski B, Wilson AC. (2009). Microbial community gene expression within colonies of the diazotroph, *Trichodesmium*, from the Southwest Pacific Ocean. ISME J 3: 1286–1300. 19571897 10.1038/ismej.2009.75

[bib25] Hmelo L, Van Mooy B, Mincer T. (2012). Characterization of bacterial epibionts on the cyanobacterium *Trichodesmium*. Aquat Microb Ecol 67: 1–14.

[bib26] Hmelo LR, Mincer TJ, Van Mooy BAS. (2011). Possible influence of bacterial quorum sensing on the hydrolysis of sinking particulate organic carbon in marine environments. Environ Microbiol Rep 3: 682–688. 23761357 10.1111/j.1758-2229.2011.00281.x

[bib27] Hong KW, Koh CL, Sam CK, Yin WF, Chan KG. (2012). Quorum quenching revisited-from signal decays to signalling confusion. Sensors 12: 4661–4696. 22666051 10.3390/s120404661 PMC3355433

[bib28] Hyatt D, Chen G-L, Locascio PF, Land ML, Larimer FW, Hauser LJ. (2010). Prodigal: prokaryotic gene recognition and translation initiation site identification. BMC Bioinform 11: 1–11. 10.1186/1471-2105-11-119 PMC2848648 20211023

[bib29] Karl DM, Beversdorf L, Orkman KMBJ, Church MJ, Martinez A, DeLong EF. (2008). Aerobic production of methane in the sea. Nat Geosci 1: 473–478.

[bib30] Krishnamurthy A, Moore JK, Zender CS, Luo C. (2007). Effects of atmospheric inorganic nitrogen deposition on ocean biogeochemistry. J Geophys Res 112: G02019.

[bib31] Krupke A, Hmelo LR, Ossolinski JE, Mincer TJ, Van Mooy BAS. (2016). Quorum sensing plays a complex role in regulating the enzyme hydrolysis activity of microbes associated with sinking particles in the ocean. Front Mar Sci 3: 1–9.

[bib32] Lomas MW, Burke AL, Lomas DA, Bell DW, Shen C, Dyhrman ST et al. (2010). Sargasso Sea phosphorus biogeochemistry: an important role for dissolved organic phosphorus (DOP). Biogeosciences 7: 695–710.

[bib33] Luo H, Benner R, Long RA, Hu J. (2009). Subcellular localization of marine bacterial alkaline phosphatases. Proc Natl Acad Sci USA 106: 21219–21223. 19926862 10.1073/pnas.0907586106 PMC2795515

[bib34] Mahaffey C, Reynolds S, Davis CE, Lohan MC. (2014). Alkaline phosphatase activity in the subtropical ocean: insights from nutrient, dust and trace metal addition experiments. Front Mar Sci 1: 1–13.

[bib35] Metcalf WW, van der Donk WA. (2009). Biosynthesis of phosphonic and phosphinic acid natural products. Annu Rev Biochem 78: 65–94. 19489722 10.1146/annurev.biochem.78.091707.100215 PMC2729427

[bib36] Metcalf WW, Wolfe RS. (1998). Molecular genetic analysis of phosphite and hypophosphite oxidation by *Pseudomonas stutzeri* WM88. J Bacteriol 180: 5547–5558. 9791102 10.1128/jb.180.21.5547-5558.1998 PMC107611

[bib37] Miller MB, Bassler BL. (2001). Quorum sensing in bacteria. Annu Rev Microbiol 55: 165–199. 11544353 10.1146/annurev.micro.55.1.165

[bib38] Momper LM, Reese BK, Carvalho G, Lee P, Webb EA. (2014). A novel cohabitation between two diazotrophic cyanobacteria in the oligotrophic ocean. ISME J 9: 882–893. 10.1038/ismej.2014.186 PMC4817694 25343510

[bib39] Moore CM, Mills MM, Achterberg EP, Geider RJ, LaRoche J, Lucas MI et al. (2009). Large-scale distribution of Atlantic nitrogen fixation controlled by iron availability. Nat Geosci 2: 867–871.

[bib40] Moore CM, Mills MM, Langlois R, Milne A, Achterberg EP, La Roche J et al. (2008). Relative influence of nitrogen and phosphorus availability on phytoplankton physiology and productivity in the oligotrophic sub-tropical North Atlantic Ocean. Limnol Oceanogr 53: 291–305.

[bib41] Nausch M. (1996). Microbial activities on *Trichodesmium* colonies. Mar Ecol Prog Ser 141: 173–181.

[bib42] Oksanen J et al, Kindt R, Legendre P, O'Hara B, Simpson GL, Solymos P. (2015) Package‘Vegan’. Community Ecology Package. Version 2.2-1. 279. Available at https://github.com/vegandevs/vegan.

[bib43] Orchard ED, Webb EA, Dyhrman ST. (2009). Molecular analysis of the phosphorus starvation response in *Trichodesmium* spp. Environ Microbiol 11: 2400–2411. 19555381 10.1111/j.1462-2920.2009.01968.x

[bib44] Overbeek R, Olson R, Pusch GD, Olsen GJ, Davis JJ, Disz T et al. (2014). The SEED and the rapid annotation of microbial genomes using subsystems technology (RAST). Nucleic Acids Res 42: 206–214. 10.1093/nar/gkt1226 PMC3965101 24293654

[bib45] Paerl HW. (1994). Spatial segregation of CO2 fixation in *Trichodesmium* spp. - linkage to N2 fixation potential. J Phycol 30: 790–799.

[bib46] Patankar AV, Gonzalez JE. (2009). Orphan LuxR regulators of quorum sensing: review article. FEMS Microbiol Rev 33: 739–756. 19222586 10.1111/j.1574-6976.2009.00163.x

[bib47] Peng Y, Leung HCM, Yiu SM, Chin FYL. (2012). IDBA-UD: a *de novo* assembler for single-cell and metagenomic sequencing data with highly uneven depth. Bioinformatics 28: 1420–1428. 22495754 10.1093/bioinformatics/bts174

[bib48] Polyviou D, Hitchcock A, Baylay AJ, Moore CM, Bibby TS. (2015). Phosphite utilisation by the globally important marine diazotroph *Trichodesmium*. Environ Microbiol Rep 6: 824–830. 10.1111/1758-2229.12308 26081517

[bib49] Rao M, Smith BC, Marletta A. (2015). Nitric oxide mediates biofilm formation and symbiosis in *Silicibacter* sp. Strain TrichCH4B. MBio 6: 1–10. 10.1128/mBio.00206-15 PMC4436077 25944856

[bib50] Riebesell U, Körtzinger A, Oschlies A. (2009). Sensitivities of marine carbon fluxes to ocean change. Proc Natl Acad Sci USA 106: 20602–20609. 19995981 10.1073/pnas.0813291106 PMC2791567

[bib51] Roe KL, Barbeau K, Mann EL, Haygood MG. (2012). Acquisition of iron by Trichodesmium and associated bacteria in culture. Environ Microbiol14: 1681–1695.. 10.1111/j.1462-2920.2011.02653.x 22118517

[bib52] Rodriguez F, Lillington J, Johnson S, Timmel CR, Lea SM, Berks BC. (2014). Crystal structure of the *Bacillus subtilis* phosphodiesterase PhoD reveals and iron and calcium-containing active site. J Biol Chem 289: 30889–30899. 25217636 10.1074/jbc.M114.604892 PMC4223295

[bib53] Rouco M, Haley ST, Dyhrman ST. (2016). Microbial diversity within the *Trichodesmium* holobiont. Environ Microbiol 18: 5151–5160. 27581522 10.1111/1462-2920.13513

[bib54] Rouco M, Joy-Warren H, McGillicuddy DJ, Waterbury JJB, Dyhrman ST. (2014). *Trichodesmium* sp. clade distributions in the western North Atlantic Ocean. Limnol Oceanogr 59: 1899–1909.

[bib55] Salomon D, Klimko JA, Trudgian DC, Kinch LN, Grishin NV, Mirzaei H et al. (2015). Type VI secretion system toxins horizontally shared between marine bacteria. PLoS Pathogens 11: e1005128. 26305100 10.1371/journal.ppat.1005128 PMC4549250

[bib56] Sañudo-Wilhelmy SA, Kustka AB, Gobler CJ, Hutchins DA, Yang M, Lwiza K et al. (2001). Phosphorus limitation of nitrogen fixation by *Trichodesmium* in the central Atlantic Ocean. Nature 411: 66–69. 11333977 10.1038/35075041

[bib57] Sebastian M, Ammerman JW. (2009). The alkaline phosphatase PhoX is more widely distributed in marine bacteria than the classical PhoA. ISME J 3: 563–572. 19212430 10.1038/ismej.2009.10

[bib58] Sheridan CC, Steinberg DK, Kling GW. (2002). The microbial and metazoan community associated with colonies of *Trichodesmium* spp.: a quantitative survey. J Plankton Res 24: 913–922.

[bib59] Sohm JA, Webb EA, Capone DG. (2011). Emerging patterns of marine nitrogen fixation. Nat Rev Microbiol 9: 499–508. 21677685 10.1038/nrmicro2594

[bib60] Sohm JA, Capone D. (2006). Phosphorus dynamics of the tropical and subtropical north Atlantic: *Trichodesmium* spp. versus bulk plankton. Mar Ecol Prog Ser 317: 21–28.

[bib61] Somers E, Ptacek D, Gysegom P, Srinivasan M, Vanderleyden J. (2005). *Azospirillum brasilense* produces the auxin-like phenylacetic acid by using the key enzyme for indole-3-acetic acid biosynthesis. Appl Environ Microbiol 71: 1803–1810. 15812004 10.1128/AEM.71.4.1803-1810.2005 PMC1082559

[bib62] Spaepen S, Vanderleyden J. (2011). Auxin and plant-microbe interactions. Cold Spring Harb Perspect Biol 3: 1–13. 10.1101/cshperspect.a001438 PMC3062209 21084388

[bib63] Suzek BE, Huang H, McGarvey P, Mazumder R, Wu CH. (2007). UniRef: comprehensive and non-redundant UniProt reference clusters. Bioinformatics 23: 1282–1288. 17379688 10.1093/bioinformatics/btm098

[bib64] Tang K, Jiao N, Liu K, Zhang Y, Li S. (2012). Distribution and functions of TonB-dependent transporters in marine bacteria and environments: Implications for dissolved organic matter utilization. PLoS One 7: e41204. 22829928 10.1371/journal.pone.0041204 PMC3400609

[bib65] Teeling H, Fuchs BM, Becher D, Klockow C, Gardebrecht A, Bennke CM et al. (2012). Substrate-controlled succession of marine bacterioplankton populations induced by a phytoplankton bloom. Science 336: 608–611. 22556258 10.1126/science.1218344

[bib66] Van Mooy BAS, Hmelo LR, Sofen LE, Campagna SR, May AL, Dyhrman ST et al. (2012). Quorum sensing control of phosphorus acquisition in *Trichodesmium* consortia. ISME J 6: 422–429. 21900966 10.1038/ismej.2011.115 PMC3260506

[bib67] Van Mooy BAS, Krupke A, Dyhrman ST, Fredricks HF, Frischkorn KR, Ossolinski JE et al. (2015). Major role of planktonic phosphate reduction in the marine phosphorus redox cycle. Science 348: 783–785. 25977548 10.1126/science.aaa8181

[bib68] Vannini A, Volpari C, Gargioli C, Muraglia E, Cortese R, De Francesco R et al. (2002). The crystal structure of the quorum sensing protein TraR bound to its autoinducer and target DNA. EMBO J 21: 4393–4401. 12198141 10.1093/emboj/cdf459 PMC126196

[bib69] Walworth N, Pfreundt U, Nelson WC, Mincer T, Heidelberg JF, Fu F et al. (2015). *Trichodesmium* genome maintains abundant, widespread noncoding DNA*in situ,* despite oligotrophic lifestyle. Proc Natl Acad Sci USA 112: 4251–4256. 25831533 10.1073/pnas.1422332112 PMC4394263

[bib70] Webb EA, Jakuba RW, Moffett JW, Dyhrman ST. (2007). Molecular assessment of phosphorus and iron physiology in *Trichodesmium* populations from the western Central and western South Atlantic. Limnol Oceanogr 52: 2221–2232.

[bib71] Wu J, Sunda W, Boyle EA, Karl DM. (2000). Phosphate depletion in the Western North Atlantic Ocean. Science 289: 1998–2001. 10.1126/science.289.5480.759 10926534

[bib72] Wu Y, Simmons BA, Singer SW. (2015). MaxBin 2.0: an automated binning algorithm to recover genomes from multiple metagenomic datasets. Bioinformatics 32: 605–607. 26515820 10.1093/bioinformatics/btv638

[bib73] Yong SC, Roversi P, Lillington J, Rodriguez F, Krehenbrink M, Zeldin OB et al. (2014). A complex iron-calcium cofactor catalyzing phosphotransfer chemistry. Science 345: 1170–1173. 25190793 10.1126/science.1254237 PMC4175392

