# Supplementary figures and images for: Epibionts dominate metabolic functional potential of Trichodesmium colonies from the oligotrophic ocean
Source: ISME J. 2017 May 23;11(9):2090–101. doi: 10.1038/ismej.2017.74 (PMC5563961; doi:10.1038/ismej.2017.74)

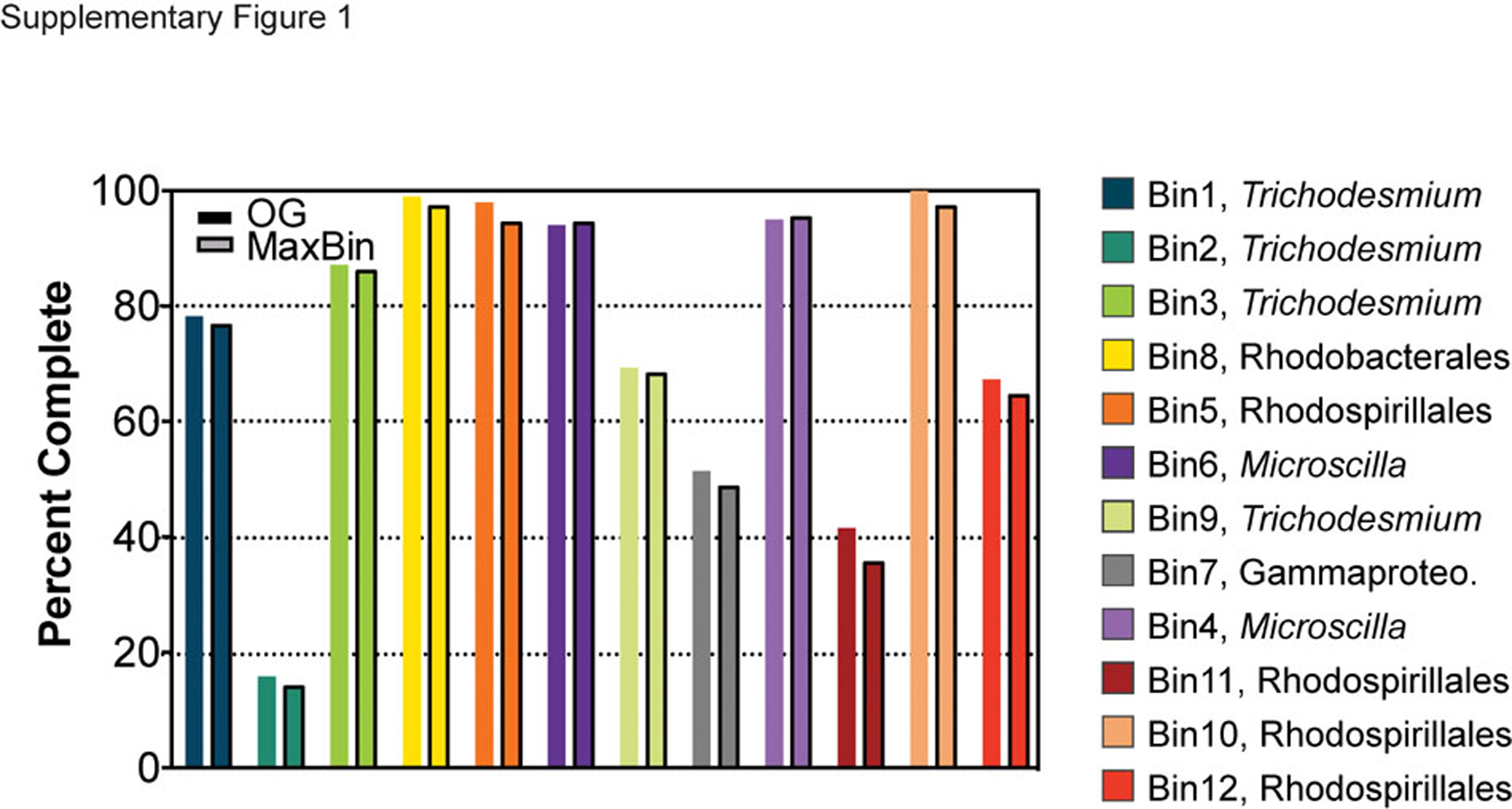

Supplement: Supplementary Figure 1 [file ismej201774x1.tif]

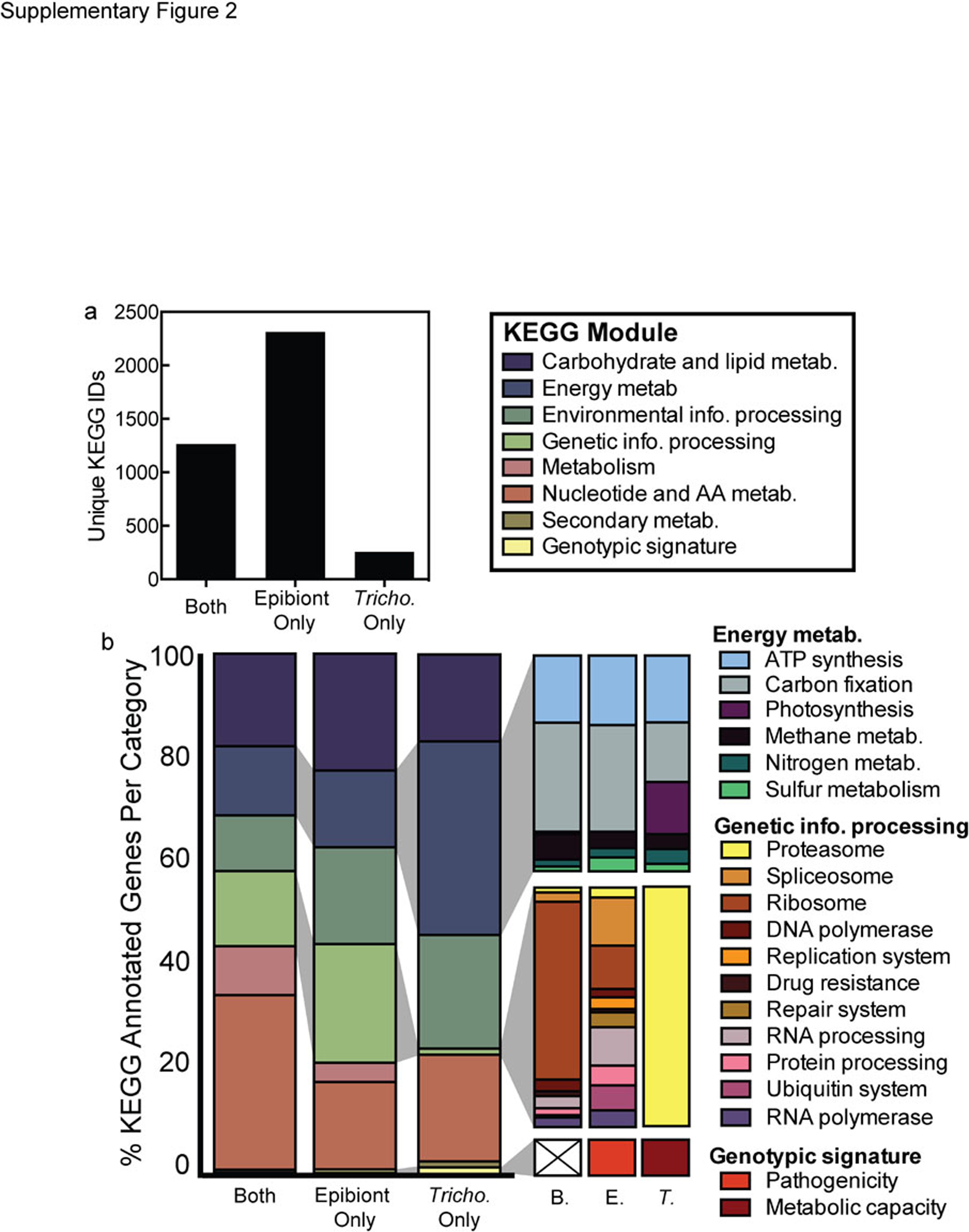

Supplement: Supplementary Figure 2 [file ismej201774x2.tif]
