# Supplementary material for: Epibionts dominate metabolic functional potential of Trichodesmium colonies from the oligotrophic ocean
Source: ISME J. 2017 May 23;11(9):2090–101. doi: 10.1038/ismej.2017.74 (PMC5563961; doi:10.1038/ismej.2017.74)
Supplement: Supplementary Table 1 [file ismej201774x3.docx]

**Supplementary Table 1.** Sequencing reads, proteins from the metagenome assembly and environmental metadata from stations in the western North Atlantic.

| **Sta-tion** | **Lat.** | **Lon.** | **Reads** | **Total prots. binned per station** | **N_2_ fixation rate (pmol colony^-1^ hr^-1^)** | **PO_4_ turnover rate (hr^-1^)** | **Sal.** | **Temp. (°C)** | **Total dissolved P (μM)** | ***Tricho.* APA (nmol P col^-1^ hr^-1^)** |
| --- | --- | --- | --- | --- | --- | --- | --- | --- | --- | --- |
| 2 | 27.9 | -65.0 | 33,044,652 | 47,177 | 4.30 | 0.03 | 36.7 | 24.0 | 0.07 | 0.124 |
| 5 | 21.2 | -64.9 | 45,080,408 | 81,064 | 8.06 | 0.07 | 36.4 | 26.4 | 0.09 | 0.146 |
| 9 | 16.5 | -57.3 | 24,522,732 | 49,198 | 39.34 | 0.06 | 36.4 | 26.4 | 0.17 | 0.288 |
| 10 | 14.0 | -55.7 | 9,067,799 | 17,975 | 14.64 | 0.05 | 34.8 | 27.1 | 0.16 | 0.087 |
| 16 | 9.9 | -58.5 | 18,492,342 | 25,845 | 16.67 | 0.06 | 34.8 | 27.5 | 0.23 | 0.134 |
| 17 | 11.9 | -59.4 | 26,590,472 | 42,773 | 20.43 | 0.09 | 35.8 | 27.4 | 0.22 | 0.200 |
