# Supplementary material for: Epibionts dominate metabolic functional potential of Trichodesmium colonies from the oligotrophic ocean
Source: ISME J. 2017 May 23;11(9):2090–101. doi: 10.1038/ismej.2017.74 (PMC5563961; doi:10.1038/ismej.2017.74)
Supplement: Supplementary Table 2 [file ismej201774x4.docx]

**Supplementary Table 2.** Frequency of key phosphorus and iron genes in the *Trichodesmium* microbiome compared to free-living bacterioplankton communities sampled from the Sargasso Sea region of the western North Atlantic.

| **Gene Family** | **% Core Microbiome^a^** | **% Free Living^b^** | **% Free Living Reference** |
| --- | --- | --- | --- |
| Phosphonate CP lyase (*PhnJ*) | 38 | 19 | Karl et al., 2008 |
|  |  | 26 | Martinez et al., 2010 |
| Siderophore/Vitamin transporters | 100 | ~18* | Tang et al., 2012 |
| Heme/Hemophores/Iron(heme) binding protein transporters | 100 | ~2* | Tang et al., 2012 |

^a^Percentage of core epibiont community genome bins (out of 8 total bins) containing the gene family

^b^Percentage of free living community calculated relative to the single copy gene *RecA*

*Values estimated from data presented in Tang et al., 2012
