# Supplementary material for: Epibionts dominate metabolic functional potential of Trichodesmium colonies from the oligotrophic ocean
Source: ISME J. 2017 May 23;11(9):2090–101. doi: 10.1038/ismej.2017.74 (PMC5563961; doi:10.1038/ismej.2017.74)
Supplement: Supplementary Table 3 [file ismej201774x5.docx]

**Supplementary Table 3.** Glycoside hydrolase (GH) orthologous groups (OGs) detected in the metagenome assemblies. Substrate specificity (target) is noted when available.

| **OG** | **Bins** | **Category** | **GH Family** | **Target** |
| --- | --- | --- | --- | --- |
| OG_1244 | 1,3,9,12 | Both | 38 | Mannose |
| OG_3154 | 3,4,6 | Both | 18 | Chitin |
| OG_11528 | 4,6 | Epibiont | NA |  |
| OG_12010 | 6 | Epibiont | 30 | Xylan |
| OG_12197 | 6 | Epibiont | 17 | 1,3;1,4-β-D-glucan |
| OG_16240 | 6 | Epibiont | 16 | β-1,4 or β-1,3 bonds in glucans and galactans |
| OG_1918 | 4,6,12 | Epibiont | 31 | Broad specificity |
| OG_20631 | Other | Epibiont | NA |  |
| OG_2728 | 4,6 | Epibiont | 3 | Broad specificity (monosaccharides) |
| OG_28509 | Other | Epibiont | NA |  |
| OG_3117 | 5,8 | Epibiont | 4 | Broad specificity (phosphorylated sugars) |
| OG_31908 | Other | Epibiont | NA |  |
| OG_3410 | 7,8,Other | Epibiont | NA |  |
| OG_3693 | 5,6 | Epibiont | 43 | Arabinose or xylose |
| OG_3765 | 4 | Epibiont | NA |  |
| OG_43634 | Other | Epibiont | NA |  |
| OG_52433 | Other | Epibiont | 18 | Chitin |
| OG_53917 | Other | Epibiont | 10 | Xylan/cellobiose |
| OG_6351 | 6 | Epibiont | 8 | Lichenin, xylan, glucan, chitin |
| OG_7475 | 4,6 | Epibiont | NA |  |
| OG_7827 | 6 | Epibiont | NA |  |
| OG_8937 | 8,11 | Epibiont | 24 | Unassigned |
| OG_9303 | Other | Epibiont | 88 | Double bonded carbons in sugars |
| OG_10713 | 3 | Tricho | 57 | Broad specificyt |
| OG_2844 | 1,3,9 | Tricho | 57 | Broad specificity |
| OG_4914 | 1,2 | Tricho | 32 | Involved in inverting sugars |
